# Supplementary material for: The class 3 PI3K coordinates autophagy and mitochondrial lipid catabolism by controlling nuclear receptor PPARα
Source: Nat Commun. 2019 Apr 5;10:1566. doi: 10.1038/s41467-019-09598-9 (PMC6451001; doi:10.1038/s41467-019-09598-9)

Supplementary Information

The class 3 PI3K coordinates autophagy and mitochondrial lipid catabolism by controlling nuclear receptor PPARα

Iershov et al.

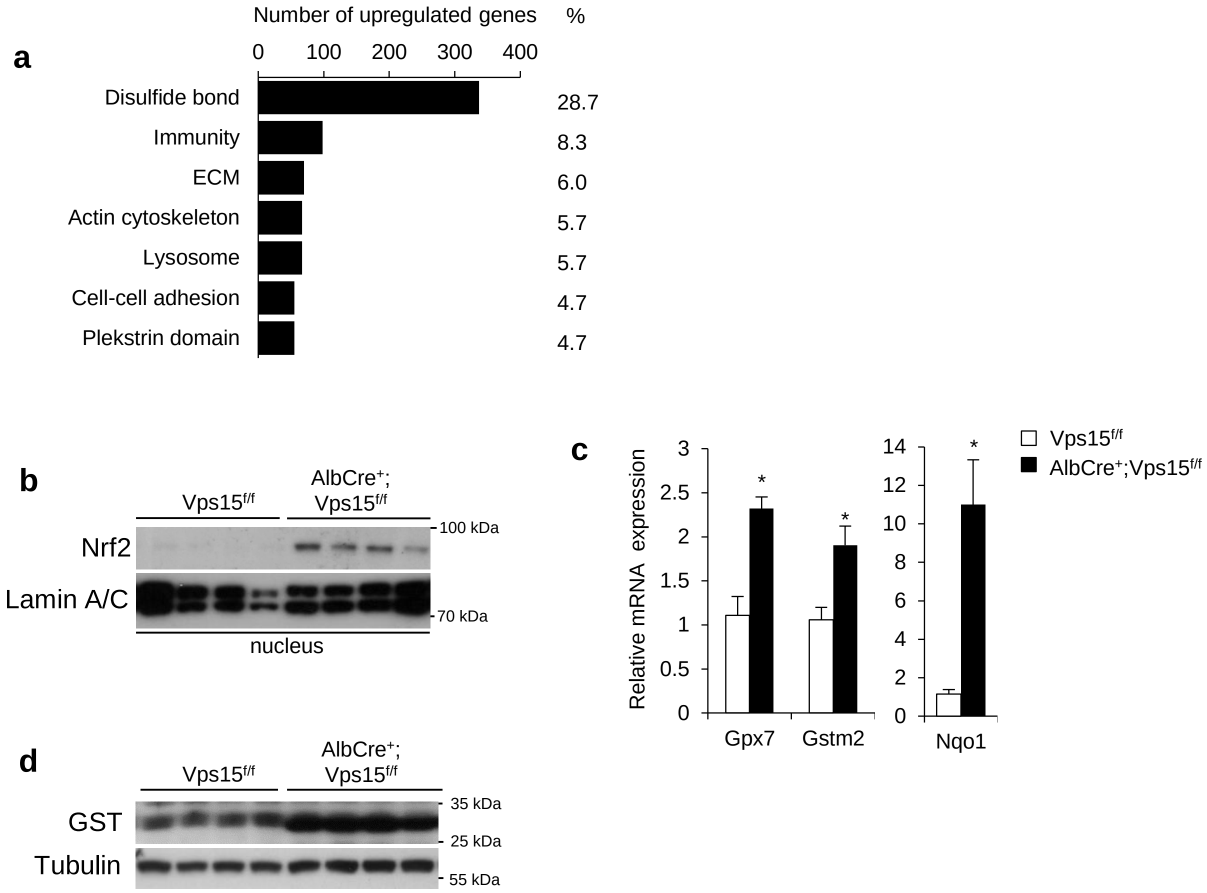

Supplementary Figure 1. Anti-oxidative stress response in livers of Vps15-LKO mice.
a Functional annotation clustering by Database for Annotation, Visualization and Integrated Discovery (DAVID) bioinformatic tool of all significantly upregulated genes in the microarray analyses of liver tissue after acute Vps15 depletion. Percentage of genes among all upregulated genes attributed to listed processes is indicated. Liver tissue was harvested ten days after transduction with adenoviral vectors expressing Cre recombinase to deplete Vps15 or GFP control protein. All significantly modified genes are listed in Supplementary Data 1. Functional annotation clustering results are listed in Supplementary Data 2. **b** Immunoblot analysis of nuclear protein liver extracts of random-fed six week old Vps15$^{f/f}$ and AlbCre^{+};Vps15$^{f/f}$ using anti-Nrf2 antibody. Immunoblot with LaminA/C antibody served as a loading control. **c** Relative transcript levels of genes implicated in anti-oxidative response in livers of control and Vps15-LKO mice analysed by RT-qPCR. Data are means ±SEM (n=8 for Vps15$^{f/f}$ n=5 for AlbCre^{+};Vps15$^{f/f}$, P<0.05 *: vs Vps15$^{f/f}$ mice, 2-tailed, unpaired Student's t test). **d** Immunoblot analysis of total protein liver extracts of random-fed six week old Vps15$^{f/f}$ and AlbCre^{+};Vps15$^{f/f}$ using anti-GST antibody. The immunoblot with anti-tubulin antibody served as a loading control.

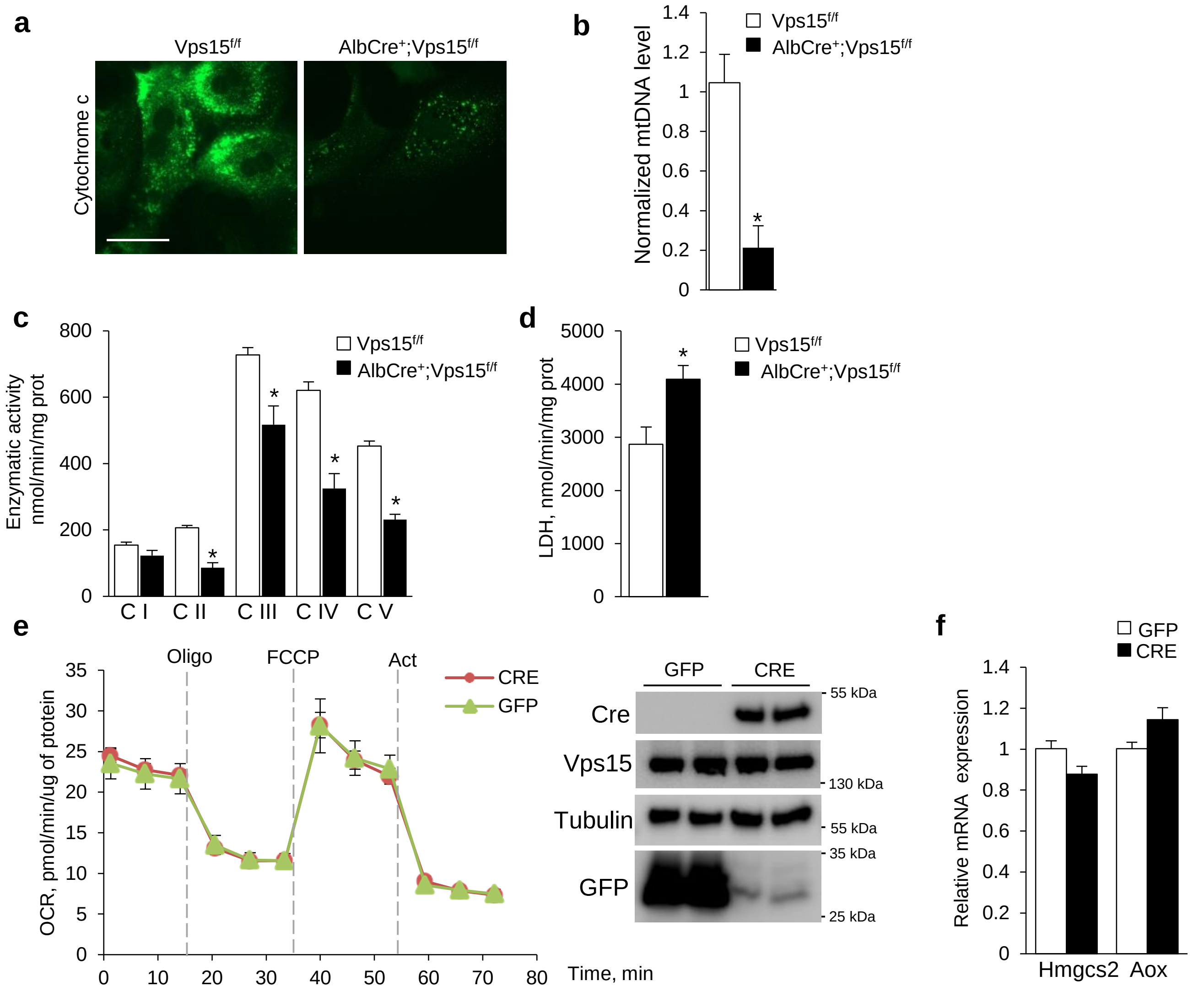

Supplementary Figure 2. Mitochondrial dysfunction in livers of Vps15-LKO mice.
a Immunofluorescent analyses of cytochrome c in control and Vps15-null primary hepatocytes. Cells were PFA fixed and stained with anti-cytochrome c antibody, secondary anti-rabbit IgG Alexa Fluor 568 antibody was used for detection (images presented in green pseudocolor). Scale bar: 50 μm. **b** Mitochondrial versus nuclear DNA was determined by quantitative PCR analysed in total DNA purified from liver tissue of random-fed six week old Vps15f/f and AlbCre+;Vps15f/f mice. Mitochondrial genome coded CO1 gene served as read-out of mtDNA and β-globin as read-out of nuclear genome (n=7 for Vps15f/f, n=5 for AlbCre+;Vps15f/f, P<0.05 *: vs Vps15f/f mice, 2-tailed, unpaired Student's t test). Enzymatic activities of the respiratory chain complexes (**c**) and lactate dehydrogenase activity (**d**) were measured in liver tissue extracts of random-fed six week old Vps15f/f and AlbCre+;Vps15f/f mice. Data are means ±SEM (n=4 for Vps15f/f and AlbCre+;Vps15f/f, P<0.05 *: vs Vps15f/f mice, 2-tailed, unpaired Student's t test). **e** The OCR measured by Seahorse Bioanalyzer in primary hepatocytes from wild-type mice transduced with adenoviral vectors expressing Cre or GFP measured 48 hours post-infection. First, OCR under basal conditions (initial rates) and then in response to sequential treatment with Oligomycin, FCCP, and Rotenone/Antimycin A is depicted. Representative experiment of three independent hepatocyte cultures is presented. Dashed lines indicate the time of the addition of each reagent. Right panel shows the control immunoblot analysis of total protein extracts of hepatocytes using indicated antibodies. **f** Relative transcript levels of indicated genes in primary hepatocytes treated as in (**e**). Data are means ±SEM, n=3.

a

Transcription factor	Enrichment	Gene overlap among downregulated genes	Sample type	Adjusted p-value	PubMed ID
RXR	0.192	384/2000	Liver	1.02E-101	22158963
PPARA	0.173	346/2000	Liver	4.06E-77	22158963
LXR	0.163	326/2000	Liver	2.33E-65	22158963
ESR1	0.241	107/444	Liver	5.92E-34	17901129
EGR1	0.123	116/944	Liver	2.22E-11	23403033
NR1I2	0.118	111/939	Liver	7.77E-10	20693526
FOXO1	0.156	54/347	Liver	1.48E-08	23066095
HNF4A	0.075	459/6083	HepG2	5.91E-07	19822575
CEBPA	0.122	72/589	Liver	7.35E-07	23403033
CLOCK	0.135	55/407	293T	1.28E-06	20551151
PPARG	0.151	41/272	3T3-L1	3.73E-06	19300518
ESRRB	0.084	121/1434	MECS	5.78E-03	18555785
FOXA2	0.076	226/2968	HepG2	5.78E-03	19822575
SREBP2	0.085	93/1095	Liver	2.70E-02	21459322
TAF7L	0.087	79/912	C3H10T1	3.68E-02	23326641

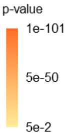

Transcription factor	Enrichment	Gene overlap among upregulated genes	Sample type	Adjusted p-value	PubMed ID
MYB	0.200	185/923	ERMYB	4.00E-35	21317192
MECOM	0.149	291/1951	KASUMI	8.68E-32	23826213
IRF8	0.144	287/2000	BMDM	1.85E-28	27001747
NCOR	0.140	280/2000	Macrophages	5.65E-26	22465074
SMRT	0.130	260/2000	Macrophages	3.31E-19	22465074
Nerf2	0.140	186/1331	Macrophages	2.10E-16	26677805
BRD4	0.120	240/2000	AML	2.13E-13	27068464
EKLF	0.133	165/1239	Erythrocyte	1.41E-12	21900194
IRF8	0.194	62/319	J774	7.93E-11	21731497
NR1H3	0.157	94/599	Atherosclerotic foam	8.79E-11	23393188
SOX2	0.113	224/1991	MEF	1.04E-09	18692474
SPI1	0.116	195/1676	GC-B	1.38E-09	22096565
NUCKS1	0.151	89/588	Hepatocytes	1.98E-09	24931609
SPI1	0.111	217/1962	Erythroleukemia	8.77E-09	22790984
SPI1	0.101	322/3198	NB4	1.22E-08	23547873

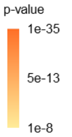

b

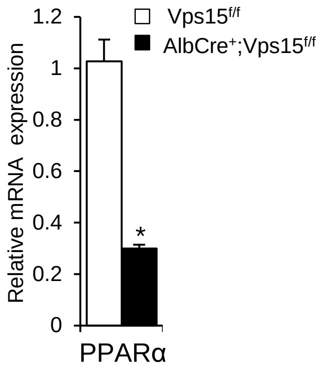

c

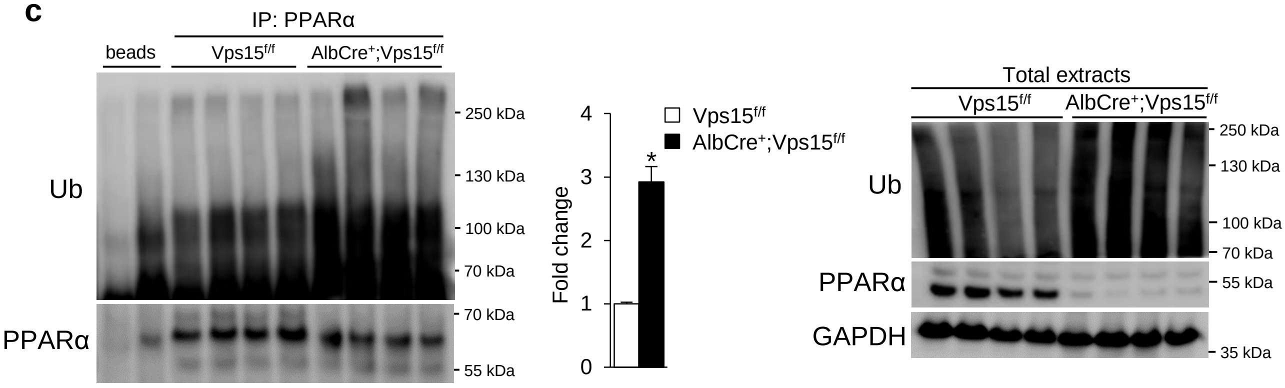

d

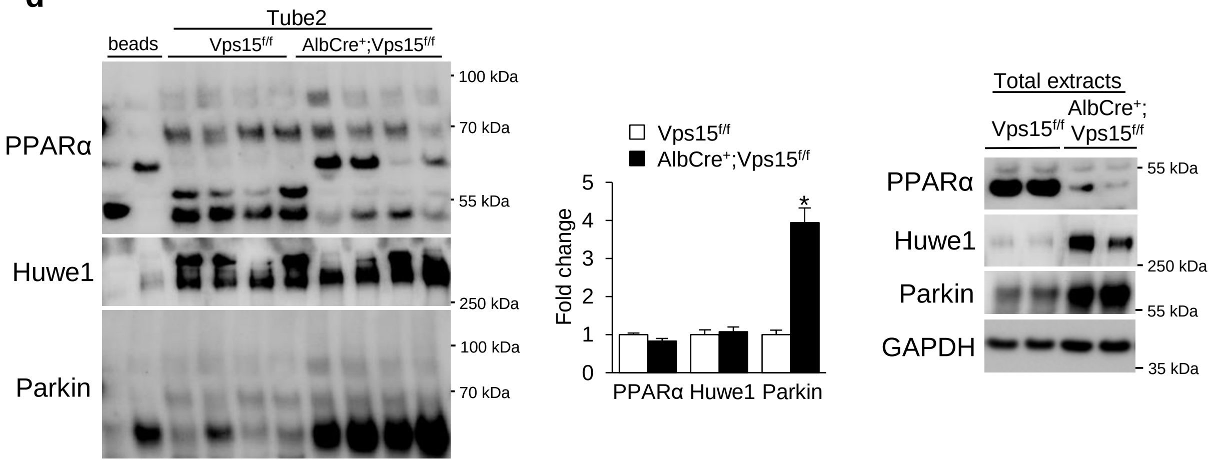

Supplementary Figure 3. PPARα protein poly-ubiquitination is induced in livers of Vps15-LKO mice.

a Bioinformatic analyses using EnrichR tool shows enrichment for transcription factors for the downregulated (upper table) and upregulated (lower table) genes in livers of Vps15-LKO mice. Adjusted p-value – p-value computed using the Fisher exact test and adjusted for multiple comparisons (the colour of the cell indicates the significance from red to yellow). **b** Relative transcript levels of PPARα gene in livers of control and Vps15-LKO mice analysed by RT-qPCR. Data are means ±SEM (n=8 for $Vps15^{f/f}$, n=5 for $AlbCre^{+}$;$Vps15^{f/f}$, P<0.05 *: vs $Vps15^{f/f}$ mice, 2-tailed, unpaired Student's t test). **c** Endogenous PPARα was pulled-down using anti-PPARα antibodies from total liver extract of six week old random-fed $Vps15^{f/f}$ and $AlbCre^{+}$;$Vps15^{f/f}$. The precipitation without antibodies served as a control of non-specific binding. Immunoblot analyses with indicated antibodies revealed increased ubiquitination of PPARα pulled-down from the livers of Vps15-LKO mice as compared to wild-type mice (left panel). Densitometric analyses of ubiquitin signal normalized to PPARα signal presented as folds over $Vps15^{f/f}$ condition (middle panel). Data are means ±SEM, n=4, P<0.05 *: vs $Vps15^{f/f}$ mice, 2-tailed, unpaired Student's t-test. Immunoblot analysis of total protein liver extracts used for immunoprecipitation with indicated antibodies is presented on right panel. Immunoblot with GAPDH antibody served as a loading control. **d** Endogenous PPARα, Huwe1 and Parkin proteins were pulled-down on Tube2 agarose from total liver extract of six week old random-fed $Vps15^{f/f}$ and $AlbCre^{+}$;$Vps15^{f/f}$ (left panel). The precipitation with control agarose beads served as a control of non-specific binding. Densitometric analyses of indicated protein levels presented as folds over $Vps15^{f/f}$ condition (middle panel). Data are means ±SEM, n=4, P<0.05 *: vs $Vps15^{f/f}$ mice, 2-tailed, unpaired Student's t-test. Immunoblot analysis of total protein liver extracts used for pull-down using indicated antibodies is presented on right panel. Immunoblot with GAPDH antibody served as a loading control.

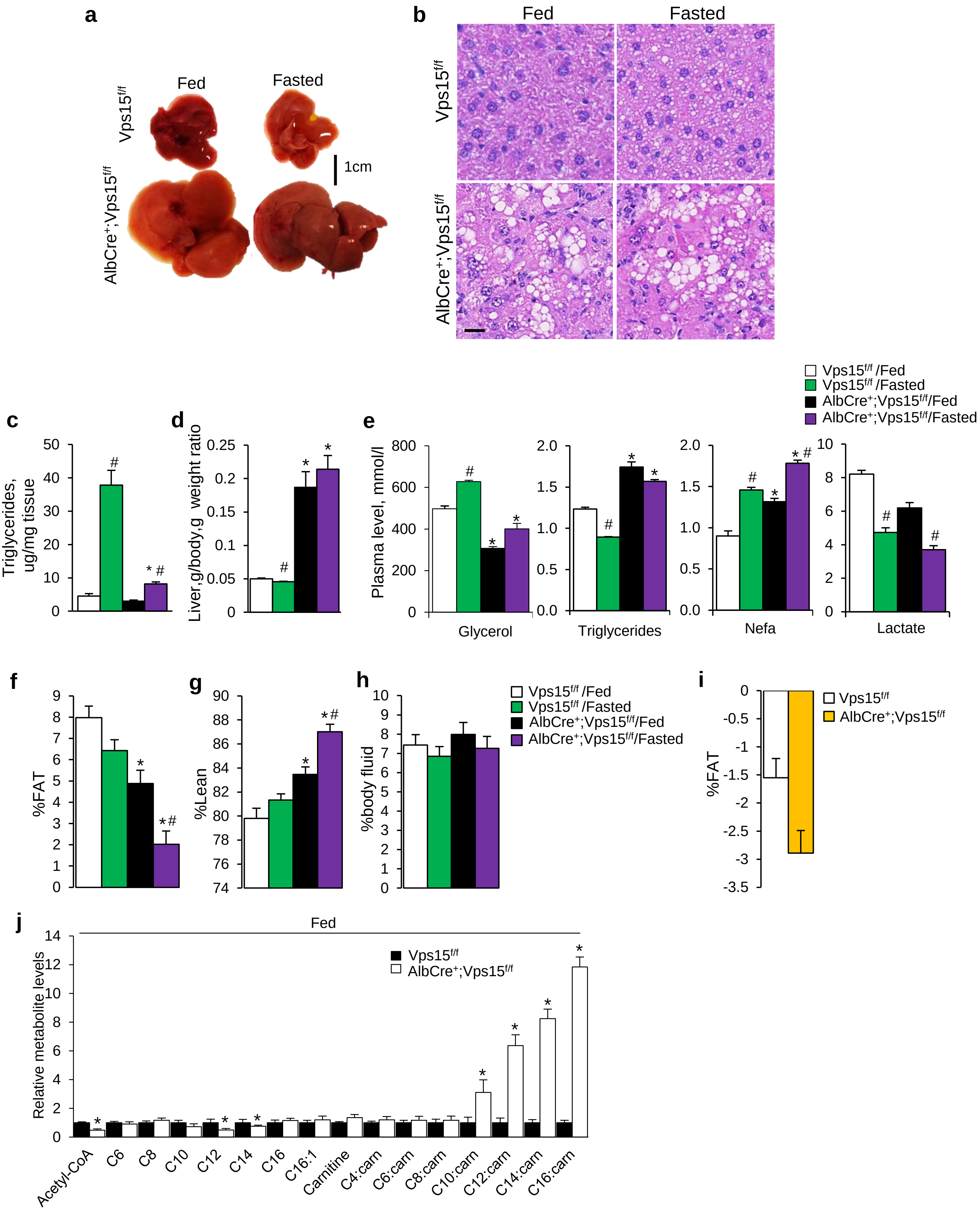

a
Fed
Fasted
Vps15f/f
AlbCre+;Vps15f/f
1cm
b
Fed
Fasted
Vps15f/f
AlbCre+;Vps15f/f
Vps15f/f /Fed
Vps15f/f /Fasted
AlbCre+;Vps15f/f/Fed
AlbCre+;Vps15f/f/Fasted
c
Triglycerides, ug/mg tissue
d
Liver,g/body,g weight ratio
e
Plasma level, mmol/l
Glycerol
Triglycerides
Nefa
Lactate
f
%FAT
g
%Lean
h
%body fluid
i
%FAT
Vps15f/f
AlbCre+;Vps15f/f
j
Fed
Relative metabolite levels
Vps15f/f
AlbCre+;Vps15f/f
Acetyl-CoA
C6
C8
C10
C12
C14
C16
C16:1
Carnitine
C4:carn
C6:carn
C8:carn
C10:carn
C12:carn
C14:carn
C16:carn

Supplementary Figure 4. Fasting phenotypes of Vps15-LKO mice.

a Macroscopic view of livers of six week old random-fed and 24-hour fasted Vps15$^{f/f}$ and AlbCre^{+};Vps15$^{f/f}$ mice. **b** HE stained liver sections of mice treated as in (**a**). Scale bar: 10 μm. **c** Triglyceride content measured enzymatically in livers of six week old random-fed or 24 hour fasted prior to sacrifice Vps15$^{f/f}$ and AlbCre^{+};Vps15$^{f/f}$ mice. Data are means ±SEM (n=6 for Vps15$^{f/f}$, n=4-5 for AlbCre^{+};Vps15$^{f/f}$, P<0.05 *: vs Vps15$^{f/f}$, #: vs fed, 2-tailed, unpaired Student's t test). **d** Liver weight to body weight ratio was determined in six week old random-fed and 24 hour fasted Vps15$^{f/f}$ and AlbCre^{+};Vps15$^{f/f}$ mice. Data are means ±SEM (Data are means ±SEM (n=6 for Vps15$^{f/f}$, n=4-5 for AlbCre^{+};Vps15$^{f/f}$, P<0.05 *: vs Vps15$^{f/f}$, #: vs fed, 2-tailed, unpaired Student's t test). **e** Plasma metabolite analyses in mice treated as in (**a**). Data are means ±SEM (Data are means ±SEM (n=6 for Vps15$^{f/f}$, n=4-5 for AlbCre^{+};Vps15$^{f/f}$, P<0.05 *: vs Vps15$^{f/f}$, #: vs fed, 2-tailed, unpaired Student's t test). Graphs showing the percentage of fat mass (**f**), lean mass (**g**) and body fluid (**h**) determined by DEXA scan of six week old random-fed and 24 hour fasted Vps15$^{f/f}$ and AlbCre^{+};Vps15$^{f/f}$ mice. Mice were fasted after the measurements in random-fed state were performed. Data are means ±SEM (n=4 for Vps15$^{f/f}$, n=5 for AlbCre^{+};Vps15$^{f/f}$, P<0.05 *: vs Vps15$^{f/f}$, #: vs fed, 2-tailed, unpaired Student's t test). **i** The graph showing the loss of fat mass upon 24 hour fasting determined by DEXA scan of six week old Vps15$^{f/f}$ and AlbCre^{+};Vps15$^{f/f}$ mice. Data are means ±SEM (n=4 for Vps15$^{f/f}$, n=5 for AlbCre^{+};Vps15$^{f/f}$, P<0.05 *: vs Vps15$^{f/f}$, 2-tailed, unpaired Student's t test). **j** Relative levels of free fatty acids, Acetyl-CoA, free carnitine and Acyl-carnitine metabolites in livers of random-fed Vps15$^{f/f}$ and AlbCre^{+};Vps15$^{f/f}$ mice measured by mass spectrometry. Data are means ±SEM (n=4, P<0.05 *: vs Vps15$^{f/f}$, 2-tailed, unpaired Student's t test).

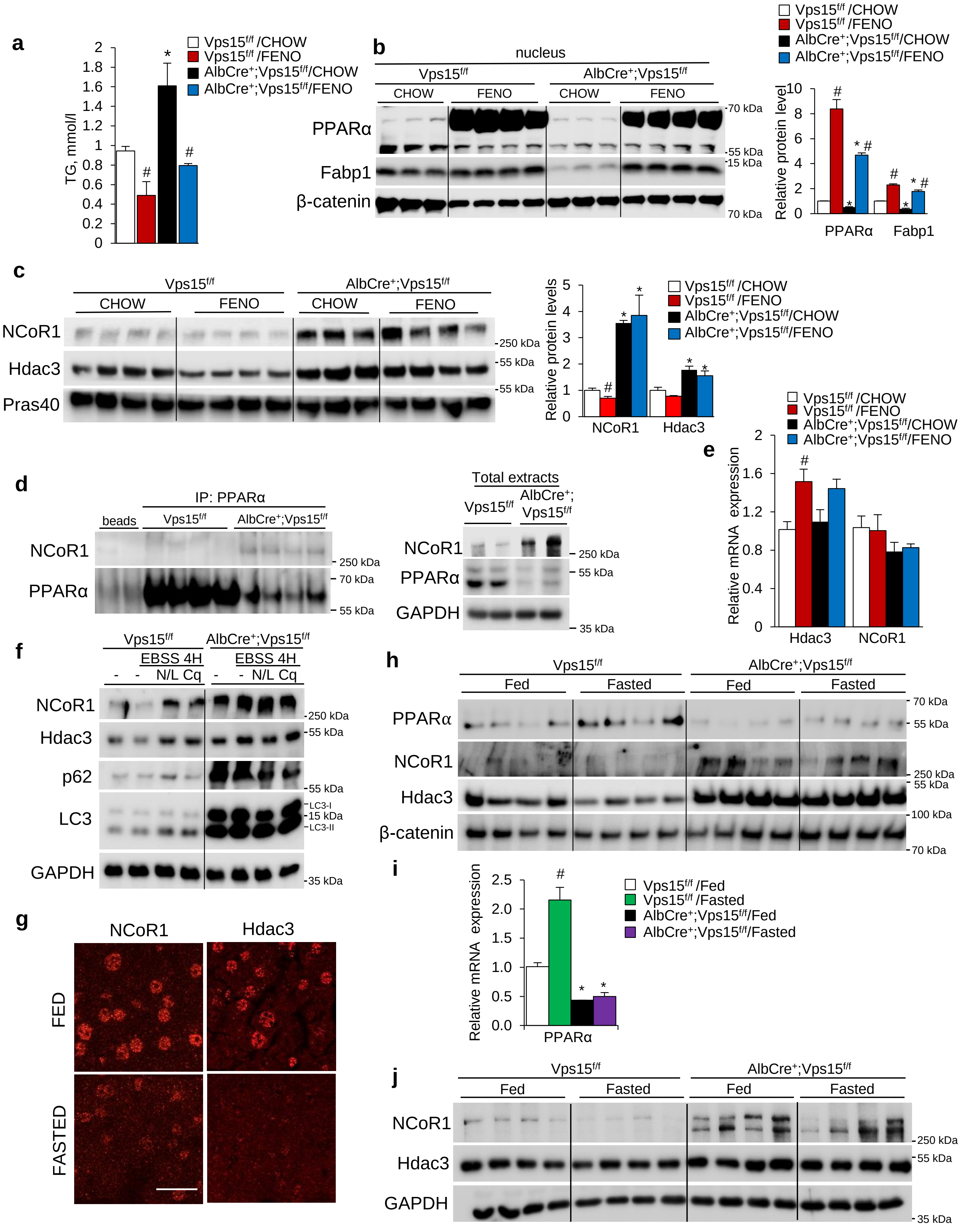
a
TG, mmol/l
Vps15f/f /CHOW
Vps15f/f /FENO
AlbCre+;Vps15f/f/CHOW
AlbCre+;Vps15f/f/FENO
b
nucleus
Vps15f/f
AlbCre+;Vps15f/f
CHOW
FENO
PPARα
Fabp1
β-catenin
70 kDa
55 kDa
15 kDa
Relative protein level
c
NCoR1
Hdac3
Pras40
250 kDa
Relative protein levels
d
IP: PPARα
beads
Total extracts
GAPDH
35 kDa
e
Relative mRNA expression
f
EBSS 4H
N/L Cq
p62
LC3
LC3-I
LC3-II
g
FED
FASTED
h
Fed
Fasted
100 kDa
i
Vps15f/f /Fed
Vps15f/f /Fasted
AlbCre+;Vps15f/f/Fed
AlbCre+;Vps15f/f/Fasted
j

Supplementary Figure 5. Fenofibrate treatment rescues PPARα activity in livers of Vps15-LKO mice.

a The biochemical analyses of triglycerides in plasma of random-fed Vps15$^{f/f}$ and AlbCre^{+};Vps15$^{f/f}$ mice that were treated for two weeks with fenofibrate incorporated in food or control chow food. Data are means ±SEM (n=4 for Vps15$^{f/f}$ chow and FENO group, n=3 for AlbCre^{+};Vps15$^{f/f}$ chow and n=4 for AlbCre^{+};Vps15$^{f/f}$ FENO group, P<0.05 *: vs Vps15$^{f/f}$, #: vs chow, 2-tailed, unpaired Student's t test). **b** Immunoblot analysis of nuclear protein liver extracts of mice treated as in (**a**) using indicated antibodies. Densitometric analyses of protein levels normalised to β-catenin levels presented as folds over Vps15$^{f/f}$-chow condition. Data are means ±SEM (n=4 for Vps15$^{f/f}$ chow and FENO group, n=3 for AlbCre^{+};Vps15$^{f/f}$ chow and n=4 for AlbCre^{+};Vps15$^{f/f}$ FENO group, P<0.05 *: vs Vps15$^{f/f}$, #: vs chow, 2-tailed, unpaired Student's t test). **c** Immunoblot analysis of total protein liver extracts of mice treated as in (**a**) using indicated antibodies. Densitometric analyses of protein levels normalised to Pras40 levels presented as folds over Vps15$^{f/f}$-chow condition. Data are means ±SEM (n=4 for Vps15$^{f/f}$ chow and FENO group, n=3 for AlbCre^{+};Vps15$^{f/f}$ chow and n=4 for AlbCre^{+};Vps15$^{f/f}$ FENO group, P<0.05 *: vs Vps15$^{f/f}$, #: vs chow, 2-tailed, unpaired Student's t test). **d** Endogenous PPARα was pulled-down using anti-PPARα antibodies from total liver extract of six week old random-fed Vps15$^{f/f}$ and AlbCre^{+};Vps15$^{f/f}$ mice. The precipitation without antibodies served as a control of non-specific binding. Immunoblot analyses with indicated antibodies revealed increased association between PPARα and NCoR1 in livers of Vps15-LKO mice compared to control mice (left panel). Immunoblot analysis of total protein liver extracts used for immunoprecipitation with indicated antibodies (right panel). Immunoblot with GAPDH antibody served as a loading control. **e** Relative mRNA expression levels of co-repressors in the livers of random-fed mice treated as in (**a**). Data are means ±SEM (Vps15$^{f/f}$ (n=6 and n=4 for chow and FENO group), AlbCre^{+};Vps15$^{f/f}$ (n=5 and n=4 for chow and FENO group), P<0.05 *: vs Vps15$^{f/f}$, #: vs chow, 2-tailed, unpaired Student's t test). **f** Immunoblot analysis of total protein liver extracts of *ex vivo* autophagic flux experiment in six week old Vps15$^{f/f}$ and AlbCre^{+};Vps15$^{f/f}$ mice using indicated antibodies. The livers were perfused with PBS solution, dissected and minced. The tissue was incubated during four hours at +37°C in complete media or in EBSS solution supplemented with lysosomal inhibitors NH_4Cl+Leupeptin (20mM+200µM) or Chloroquine (500µM). Tissue was collected by brief centrifugation and washed once with PBS before proceeding with total protein extraction. Immunoblot with anti-LC3 antibody served as a control of lysosomal inhibition (accumulation of LC3-II protein) and anti-GAPDH as a loading control. **g** Representative images of immunofluorescent analyses showing nuclear localization of NCoR1 and Hdac3 in liver tissue of wild-type mice fasted for 24 hours. Secondary anti-rabbit IgG Alexa Fluor 568 antibody was used for detection. Scale bar: 40 µm. **h** Immunoblot analysis of nuclear protein liver extracts of six week old random-fed or 24 hour fasted prior to sacrifice Vps15$^{f/f}$ and AlbCre^{+};Vps15$^{f/f}$ mice using indicated antibodies. The immunoblot with anti-β-catenin antibody served as a loading control. **i** Relative transcript levels of PPARα gene in livers of control and Vps15-LKO mice treated as in (**h**) analysed by RT-qPCR. Data are means ±SEM (n=6 for Vps15$^{f/f}$, n=4-5 for AlbCre^{+};Vps15$^{f/f}$, P<0.05 *: vs Vps15$^{f/f}$, #: vs fed, 2-tailed, unpaired Student's t test). **j** Immunoblot analysis of total protein liver extracts of six week old random-fed or 24 hour fasted prior to sacrifice Vps15$^{f/f}$ and AlbCre^{+};Vps15$^{f/f}$ mice using indicated antibodies. The immunoblot with anti-GAPDH antibody served as a loading control.

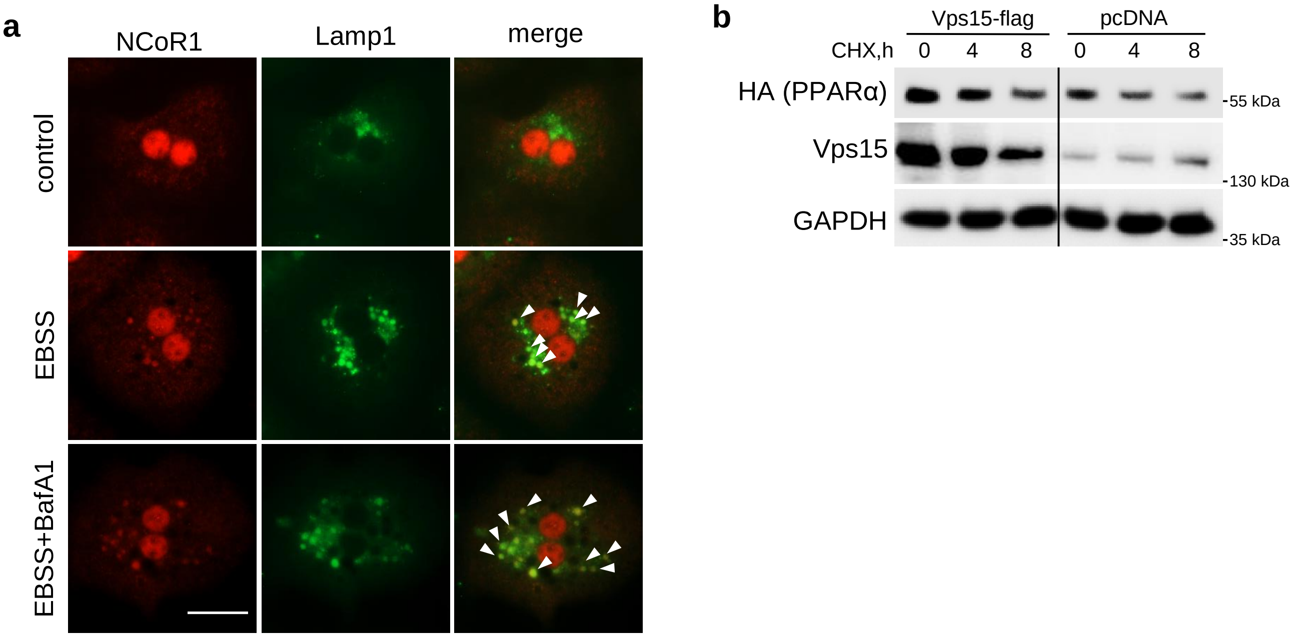

Supplementary Figure 6. PPARα regulation by Vps15.

a Immunofluorescent analyses of NCoR1 and Lamp1 proteins in control primary hepatocytes. Cells were PFA fixed and co-stained with primary antibodies, secondary anti-rabbit IgG Alexa Fluor 568 and anti-rat IgG Alexa Fluor 488 antibodies were used for detection. The white arrowheads indicate double-positive cytosolic structures. Scale bar: 40 µm. **b** Immunoblot analysis of ectopically expressed HA-tagged PPARα protein in HEK293 cells transfected with empty vector or with vector expressing Vps15-Flag. 24 hour post-transfection cells were treated for indicated times with cycloheximide (100µg/ml). Immunoblot with anti-GAPDH antibody served as a loading control.

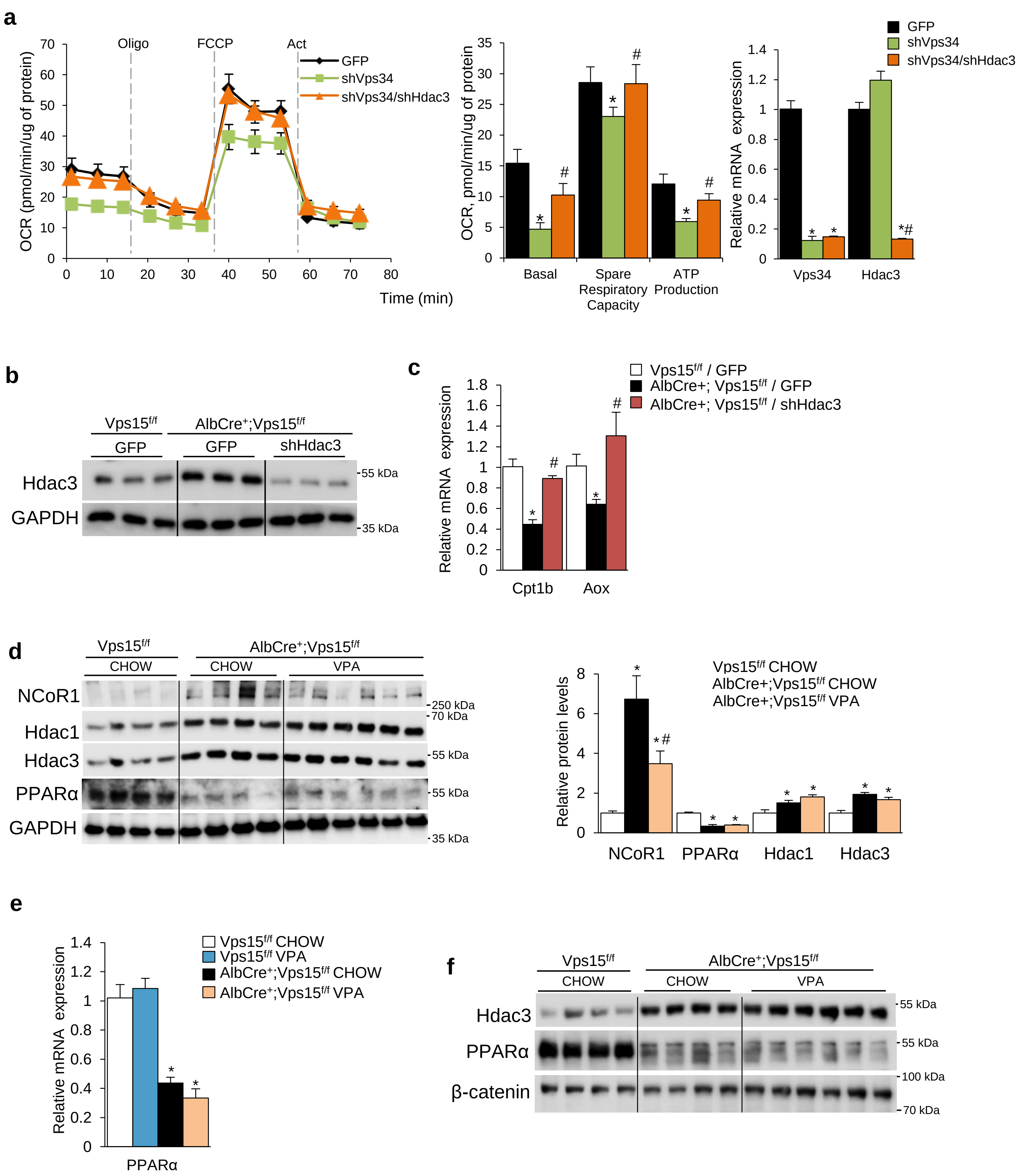
a
Oligo
FCCP
Act
GFP
shVps34
shVps34/shHdac3
OCR (pmol/min/ug of protein)
Time (min)
OCR, pmol/min/ug of protein
Basal
Spare Respiratory Capacity
ATP Production
Relative mRNA expression
Vps34
Hdac3
b
Vps15f/f
AlbCre+;Vps15f/f
GFP
GFP
shHdac3
Hdac3
GAPDH
55 kDa
35 kDa
c
Vps15f/f / GFP
AlbCre+; Vps15f/f / GFP
AlbCre+; Vps15f/f / shHdac3
Relative mRNA expression
Cpt1b
Aox
d
Vps15f/f
AlbCre+;Vps15f/f
CHOW
CHOW
VPA
NCoR1
Hdac1
Hdac3
PPARα
GAPDH
250 kDa
70 kDa
55 kDa
55 kDa
35 kDa
Vps15f/f CHOW
AlbCre+;Vps15f/f CHOW
AlbCre+;Vps15f/f VPA
Relative protein levels
NCoR1
PPARα
Hdac1
Hdac3
e
Vps15f/f CHOW
Vps15f/f VPA
AlbCre+;Vps15f/f CHOW
AlbCre+;Vps15f/f VPA
Relative mRNA expression
PPARα
f
Vps15f/f
AlbCre+;Vps15f/f
CHOW
CHOW
VPA
Hdac3
PPARα
β-catenin
55 kDa
55 kDa
100 kDa
70 kDa

Supplementary Figure 7. Hdac inhibition partially restores PPARα activity.

a The oxygen consumption rate measured by SeaHorse Bioanalyzer in primary hepatocytes isolated from wild-type mice 48 hours post-transduction with adenoviral vectors expressing GFP, shRNAVps34 or shRNAVps34+shRNAHdac3 under basal conditions (initial rates) and in response to sequential treatment with Oligomycin (respiration associated with ATP production), FCCP (maximal respiration), and Rotenone/Antimycin A (non-mitochondrial respiration). Dashed lines indicate the time of the addition of each reagent. Representative experiment of three independent hepatocyte cultures is presented. Quantification of basal respiration, ATP production and maximal respiratory capacity are shown on the graphs (middle panel). Relative mRNA expression levels of *Vps34* and *Hdac3* showing the level of knockdown of transcrips of indicated genes is shown on right panel. Data are means ±SEM ($P<0.05$ *: vs GFP-infected cells, #: vs shRNAVps34-infected cells, 2-tailed, unpaired Student's t test). **b** Immunoblot analysis of Hdac3 levels in total protein extracts of primary hepatocytes 36 hours post-transduction with adenoviral vectors expressing shRNAHdac3 or GFP as a control. Immunoblot with anti-GAPDH antibody served as a loading control. **c** Relative transcript levels of indicated genes in primary hepatocytes treated as in (**b**). Data are means ±SEM ($n=3$, $P<0.05$ *: vs GFP, unpaired Student's t test). **d** Immunoblot analysis of total protein liver extracts of six week old random-fed $Vps15^{f/f}$ and $AlbCre^{+}$;$Vps15^{f/f}$ mice that were treated for two weeks with VPA incorporated in food or control chow food using indicated antibodies. Densitometric analyses of protein levels normalised to GAPDH levels presented as folds over $Vps15^{f/f}$-chow condition. Data are means ±SEM ($n=4$ for $Vps15^{f/f}$ and $AlbCre^{+}$;$Vps15^{f/f}$ chow, $n=6$ $AlbCre^{+}$;$Vps15^{f/f}$ VPA, $P<0.05$ *: vs $Vps15^{f/f}$, #: vs chow, 2-tailed, unpaired Student's t test). **e** Relative mRNA expression levels of PPARα in the livers of mice treated as in (**d**). Data are means ±SEM ($n=6$ for $Vps15^{f/f}$ and $AlbCre^{+}$;$Vps15^{f/f}$ chow, $n=5$ $Vps15^{f/f}$ VPA, $n=6$ $AlbCre^{+}$;$Vps15^{f/f}$ VPA, $P<0.05$ *: vs $Vps15^{f/f}$, #: vs chow, 2-tailed, unpaired Student's t test). **f** Immunoblot analysis of nuclear extracts from the liver tissue of mice treated as in (**d**) using indicated antibodies. The immunoblot with anti-β-catenin antibody served as a loading control.

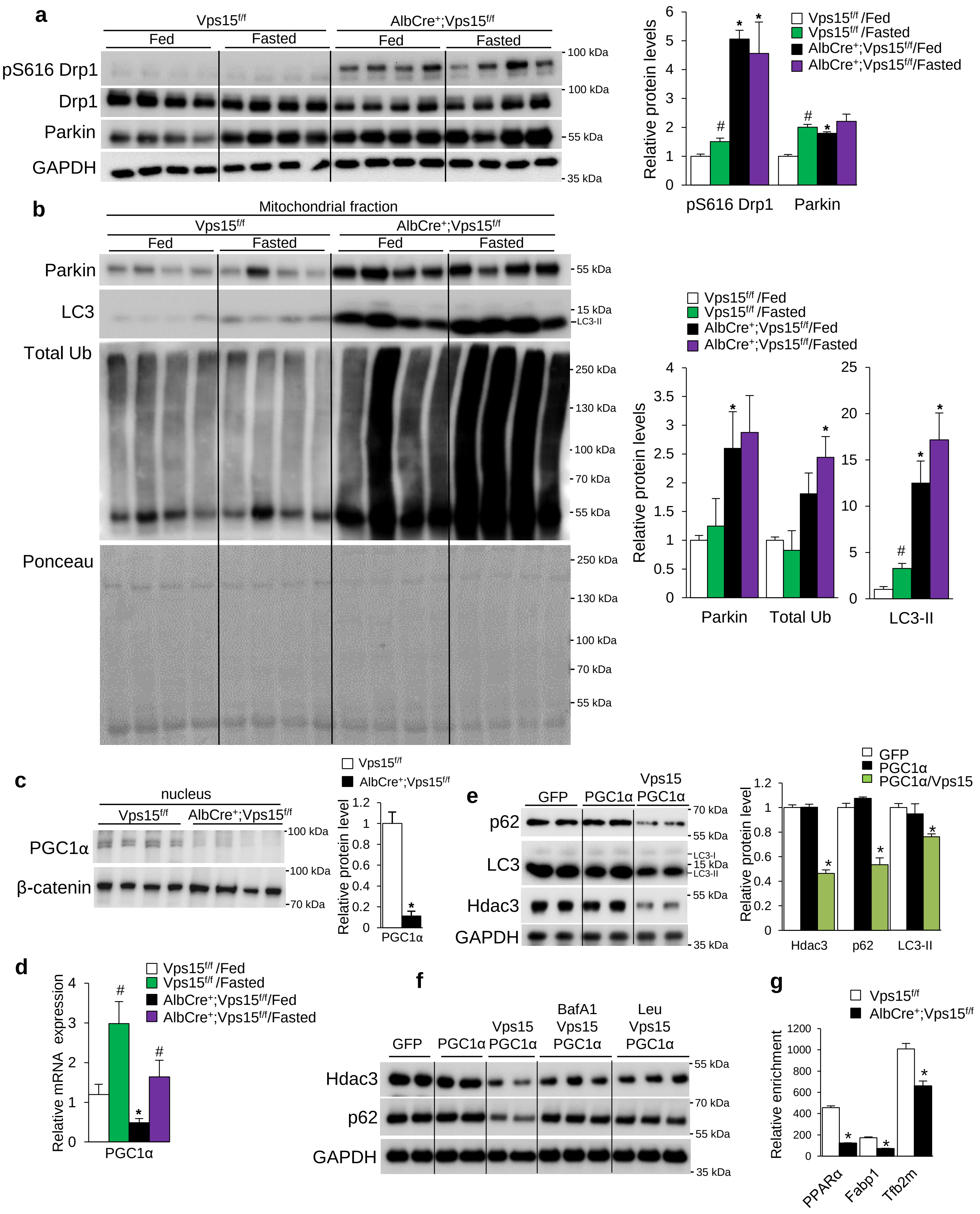

a
Vps15f/f
AlbCre+;Vps15f/f
Fed
Fasted
Fed
Fasted
pS616 Drp1
Drp1
Parkin
GAPDH
100 kDa
100 kDa
55 kDa
35 kDa
Relative protein levels
Vps15f/f /Fed
Vps15f/f /Fasted
AlbCre+;Vps15f/f/Fed
AlbCre+;Vps15f/f/Fasted
pS616 Drp1
Parkin
b
Mitochondrial fraction
Vps15f/f
AlbCre+;Vps15f/f
Fed
Fasted
Fed
Fasted
Parkin
LC3
Total Ub
Ponceau
55 kDa
15 kDa
LC3-II
250 kDa
130 kDa
100 kDa
70 kDa
55 kDa
250 kDa
130 kDa
100 kDa
70 kDa
55 kDa
Vps15f/f /Fed
Vps15f/f /Fasted
AlbCre+;Vps15f/f/Fed
AlbCre+;Vps15f/f/Fasted
Relative protein levels
Parkin
Total Ub
LC3-II
c
nucleus
Vps15f/f
AlbCre+;Vps15f/f
PGC1α
β-catenin
100 kDa
100 kDa
70 kDa
Vps15f/f
AlbCre+;Vps15f/f
Relative protein level
PGC1α
e
GFP
PGC1α
Vps15 PGC1α
p62
LC3
Hdac3
GAPDH
70 kDa
55 kDa
LC3-I
15 kDa
LC3-II
55 kDa
35 kDa
GFP
PGC1α
PGC1α/Vps15
Relative protein level
Hdac3
p62
LC3-II
d
Vps15f/f /Fed
Vps15f/f /Fasted
AlbCre+;Vps15f/f/Fed
AlbCre+;Vps15f/f/Fasted
Relative mRNA expression
PGC1α
f
GFP
PGC1α
Vps15 PGC1α
BafA1 Vps15 PGC1α
Leu Vps15 PGC1α
Hdac3
p62
GAPDH
55 kDa
70 kDa
55 kDa
35 kDa
g
Vps15f/f
AlbCre+;Vps15f/f
Relative enrichment
PPARα
Fabp1
Tfb2m

Supplementary Figure 8. Defective mitochondrial maintenance and PGC1α expression in Vps15 mutants.

Immunoblot analyses of total liver extracts (**a**) and mitochondrial fraction (**b**) of six week old random-fed or 24 hour fasted prior to sacrifice $Vps15^{f/f}$ and $AlbCre^{+}$;$Vps15^{f/f}$ mice. Plotted densitometric analyses of normalized phospho-Ser616 Drp1 to total Drp1 and Parkin to GAPDH levels in total extracts; and Parkin, total ubiquitin and LC3-II levels to total ponceau signal presented as folds over $Vps15^{f/f}$ fed condition (right panel). Data are means ±SEM (n=4 for $Vps15^{f/f}$ and $AlbCre^{+}$;$Vps15^{f/f}$, P<0.05 *: vs $Vps15^{f/f}$, #: vs fed, 2-tailed, unpaired Student's t test). **c** Immunoblot analysis of nuclear protein liver extracts of random-fed six week old $Vps15^{f/f}$ and $AlbCre^{+}$;$Vps15^{f/f}$ using indicated antibodies. Densitometric analyses of PGC1α protein levels normalised to β-catenin levels presented as folds over $Vps15^{f/f}$ condition (right panel). Data are means ±SEM (n=4 for $Vps15^{f/f}$ and $AlbCre^{+}$;$Vps15^{f/f}$, P<0.05 *: vs $Vps15^{f/f}$, 2-tailed, unpaired Student's t test). **d** Relative transcript levels of PGC1α in the livers of six week old random-fed or 24 hour fasted prior to sacrifice $Vps15^{f/f}$ and $AlbCre^{+}$;$Vps15^{f/f}$ mice. Data are means ±SEM (n=4 for $Vps15^{f/f}$, n=6 for $AlbCre^{+}$;$Vps15^{f/f}$, P<0.05 *: vs $Vps15^{f/f}$, #: vs fed, 2-tailed, unpaired Student's t test). **e** Immunoblot analysis using indicated antibodies of total protein extracts of primary hepatocytes 36 hours post-transduction with indicated adenoviral vectors. Densitometric analyses of protein levels normalised to GAPDH levels presented as folds over GFP-infected cells. Data are means ±SEM (n=3 independent hepatocyte cultures, P<0.05 *: vs GFP, 2-tailed, unpaired Student's t test). **f** Immunoblot analysis of total protein extracts of primary hepatocytes transduced with indicated adenoviral vectors. 12 hours post-transduction PGC1α+Vps15 infected cells were treated for additional 24 hours with vehicle, Leupeptin or BafA1. All cells were collected at the same time and the cytosolic extracts were prepared for further immunoblot analyses. Immunoblot with anti-GAPDH antibody served as a loading control. **g** Livers of six week old random-fed $Vps15^{f/f}$ and $AlbCre^{+}$;$Vps15^{f/f}$ were subjected to chromatin immunoprecipitation with anti-H3K27Ac antibody followed by qPCR analyses using primers nested around PPARα-responsive elements in promoter regions of indicated genes. Data are presented as relative enrichment in anti-H3K27Ac samples to immunoprecipitation with nonspecific IgG. Data are means ±SEM, n=4, P<0.05 *: vs wild-type mice, 2-tailed, unpaired Student's t-test.

Supplementary Table 1. Primer sequences used for qPCR analyses

ChIP

Primer name	Sequence
Tfam_ppre1_S	CAACTTGTTTGAGTTGGTTCTC
Tfam_ppre1_AS	AATAGAAAGGAATGAGGGTTCC
Tfam_ppre2_S	GACCTAGCCAAACCAAAGAG
Tfam_ppre2_AS	CTTCCTGTCACCTTCTACCT
Tfb2m_ppre1_S	GGACCTAGAATGCAAGATG
Tfb2m_ppre1_AS	GGTGTTTGAGGTGGACTTT
Tfb2m_ppre2_S	AAACAGGAGATCCGAGAGG
Tfb2m_ppre2_AS	CGGATGCCCAGGTAAGA
Ppara_ppre_S	CAGTAGGCAAGTAGGGAATG
Ppara_ppre_AS	AAACCCACCTTTGCTGAT
FABP1_ppre_S	ACAAACCTCTGCCTTGCCCA
FABP1_ppre_AS	CAACGAGGCTTCCTTTCCACA

qPCR

Primer name	Sequence
Aox_S	GGGAGTGCTACGGGTTACATG
Aox_AS	CCGATATCCCCAACAGTGATG
ATP6_S	CCTTCCACAAGGAACTCCAATTTCAC
ATP6_AS	CTAGAGTAGCTCCTCCGATTAGGTG
ATP8_S	ATGCCACAACTAGATACATCAACATG
ATP8_AS	GTGATTTTGGTGAAGGTGCCAGTGG
Cpt1_S	TGGGACTGGTCGATTGCATC
Cpt1_AS	TCAGGGTTTGTCGGAAGAAGAA
Cpt2_S	CAGCACAGCATCGTACCCA
Cpt2_AS	TCCCAATGCCGTTCTCAAAAT
CYTB_S	CTACTGTTCGCAGTCATAGCCAC
CYTB_AS	CCAATATATGGGATGGCTGATAGGAG
Fabp1_S	TCCGCAATGAGTTCACCCTG
Fabp1_AS	CAGCTTGACGACTGCCTTGA
Gstm2_S	TTCTTCAGGCCCTCAAAGC
Gstm2_AS	TGTCCTTGATCAACACCGAA
Gpx7_S	CTGTGAAGCCACATTCGCTA
Gpx7_S	GACTTCAAGGCGGTCAACAT
Hdac3_S	ATGCCTTCAACGTGGGTGAT
Hdac3_AS	CAGAAGCCAGAGGCCTCAAA
Hmgcl_S	AACTGGTGCAGAAATCTCTAGC
Hmgcl_AS	GGTTGAATAGCTCAGAACTAGCC
Hmgcs2_S	GAAGAGAGCGATGCAGGAAAC
Hmgcs2_AS	GTCCACATATTGGGCTGGAAA
Lcad_S	TTTCCGGGAGAGTGTAAGGA
Lcad_AS	ACTTCTCCAGCTTTCTCCCA
Mcad_S	GATGCATCACCCTCGTGTAAC
Mcad_AS	AAGCCCTTTTCCCCTGAA
Mfn2_S	CTCCCTTCTTGCCACTGAGG
Mfn2_AS	ACCACCTGGAGCAAACTAGC
MTCO1_S	GCAGGAGCATCAGTAGACCTAAC
MTCO1_AS	GGAGTTTGATACTGTGTTATGGCTGG
MTCO2_S	CATAGGGCACCAATGATACTGAAGC
MTCO2_AS	GCAGAACGACTCGGTTATCAACTTC
Ncor1_S	TGCGTCAGCTTTCTGTGATTCCACC
Ncor1_AS	TGATTTCTGCCTCTGCGTTTTCCAT

ND1_S	CACCTACCCTATCACTCACACTAGC
ND1_AS	GGCTCATCCTGATCATAGAATGGAG
ND2_S	GCATGAGGAGGACTTAACCAAACAC
ND2_AS	GAGGTTGAGTAGAGTGAGGGATGG
ND4_S	CACAACACACACCTTAGACGCTTC
ND4_AS	CAGCAATTGGAGCTTCAACATGGG
Nqo1_S	AGCGTTCGGTATTACGATCC
Nqo1_AS	AGTACAATCAGGGCTCTTCTCG
Pex5_S	GTGGGCAGCAGAGTTTATACAG
Pex5_AS	CTCCCTCCTCAAGTCGATGC
Polg_S	GCAGGAACAGTTAGTGGTGGG
Polg_AS	GCTCATAGTATCGAGAAAACGCA
Polrmt_S	TGGGCGCAAAAGCTAGAGG
Polrmt_AS	GTGAAGGGTCCAGAACTCCTG
Tert1_S	CTAGCTCATGTGTCAAGACCCTCTT
Tert1_AS	GCCAGCACGTTTCTCTCGTT
Tfam_S	ATTCCGAAGTGTTTTTCCAGCA
Tfam_AS	TCTGAAAGTTTTGCATCTGGGT
Tfb1m_S	CGGGAGATCATTAAGTTGTTCGG
Tfb1m_AS	GCCCAGGACCCACTTCATAAA
Tfb2m_S	CGGAAACTGATAGCGACTCCT
Tfb2m_AS	CACACTAAATGACGACCAAGGTT
Vlcad_S	CCGGTTCTTTGAGGAAGTGAA
Vlcad_AS	AGTGTCGTCCTCCACCTTCTC
Vps15_S	CCTGGTGGTTGTGAAGGTCT
Vps15_AS	AGCGCTTCTCGATGTTGTTT
Vps34_S	CCTGGACATCAACGTGCAG
Vps34_AS	TGTCTCTTGGTATAGCCCAGAAA
PPARα_S	AGAGCCCCATCTGTCCTCTC
PPARα_AS	ACTGGTAGTCTGCAAAACCAAA
Pinin_S	ACCTGGAAGGGGCAGTCAGTA
Pinin_AS	ATCATCGTCTTCTGGGTCGCT
b-actin_S	GTGACGTTGACATCCGTAAAGA
b-actin_AS	GCCGGACTCATCGTACTCC
Cyclophilin_S	CAGGTCCTGGCATCTTGTCC
Cyclophilin_AS	TTGCTGGTCTTGCCATTCCT
Ef1a1_S	GCAGCAACAATCAGGACAGC
Ef1a1_AS	GACATCTCCCTGTGGAAATTCG
Gapdh_S	TGACCACAGTCCATGCCATC
Gapdh_AS	GACGGACACATTGGGGGTAG
18s_S	AGTCCCTGCCCTTTGTACACA
18s_AS	CGATCCGAGGGCCTCACTA
26s_S	CCTGCTATCTCAGAACCGAGT
26s_AS	CCATGAGGTATTGTTCGAGGGAA
H2az_S	AAGCGTATCACCCCTCGTCA
H2az_AS	AGCGATTTGTGGATGTGTGG
36b4_S	TCCAGGCTTTGGGCATCA
36b4_AS	CTTTATCAGCTGCACATCACTCAGA

Uncropped blots Figure 1e

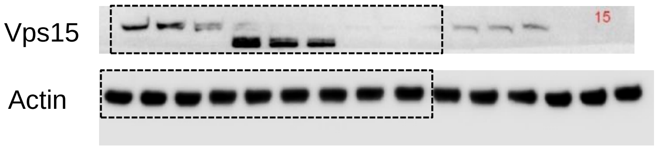

Uncropped blots Figure 2f

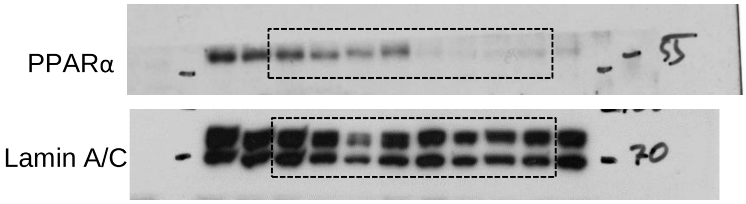

Uncropped blots Figure 3

3b

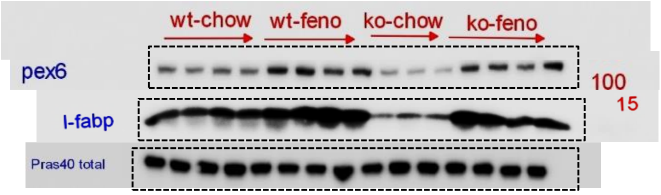

3e

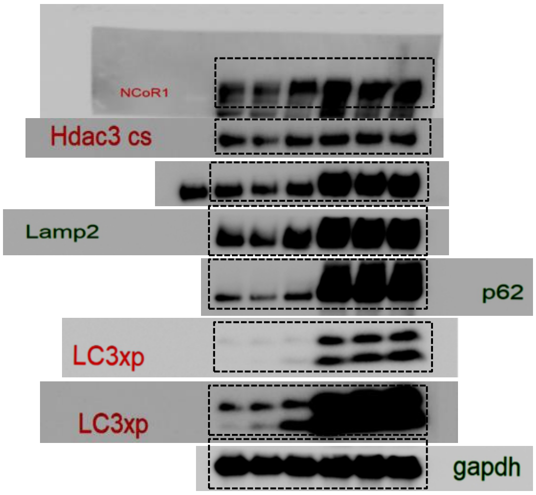

3f

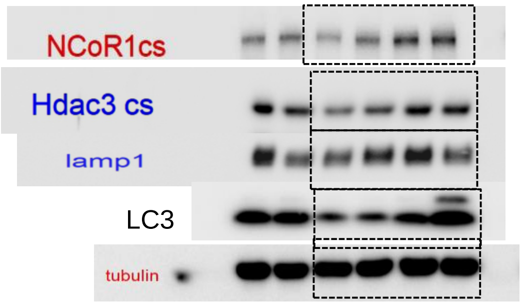

3g

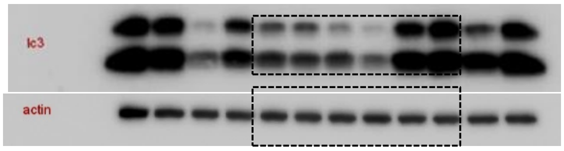

Uncropped blots Figure 4

4b

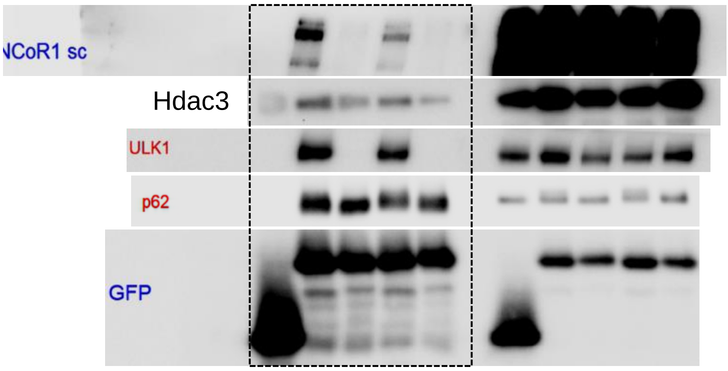

4c

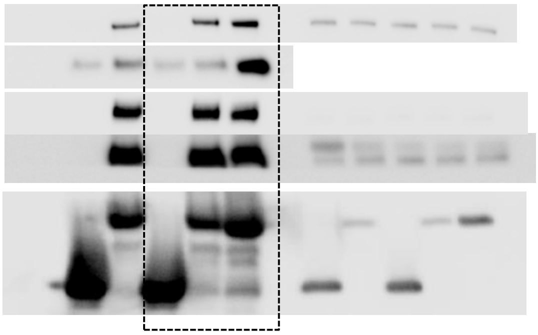

4d

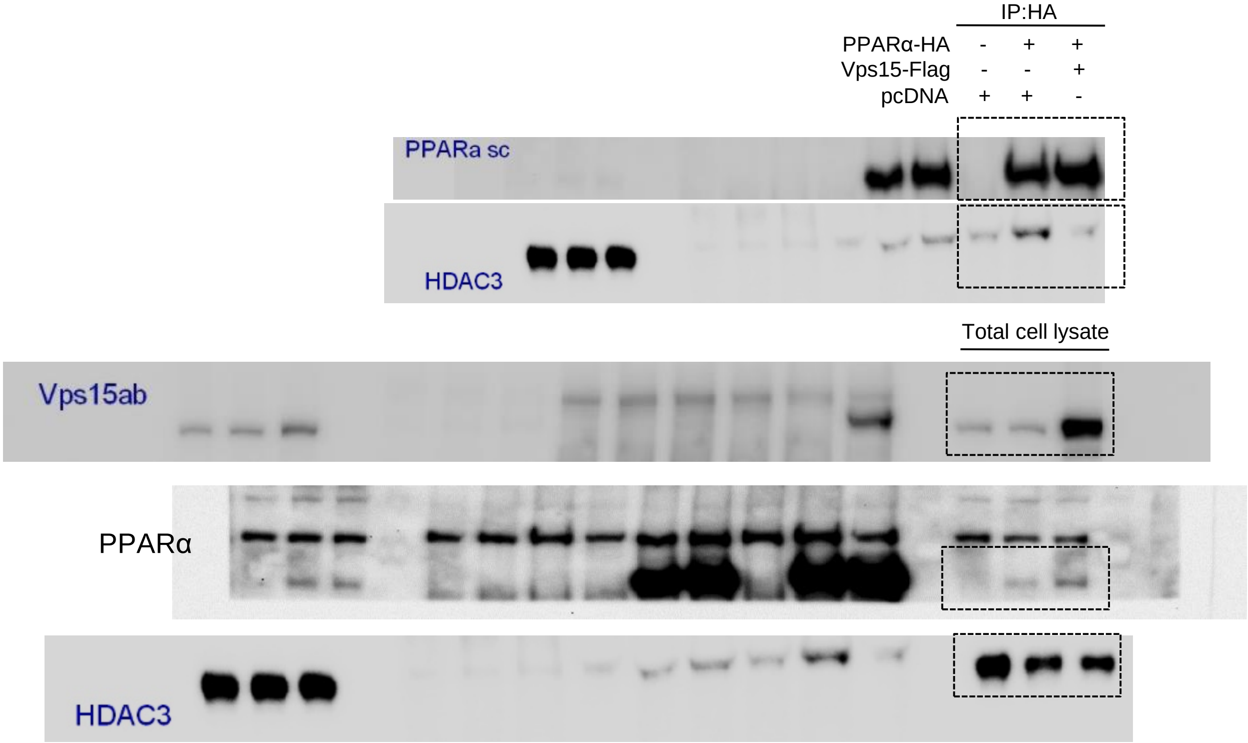

Uncropped blots Figure 5

5b

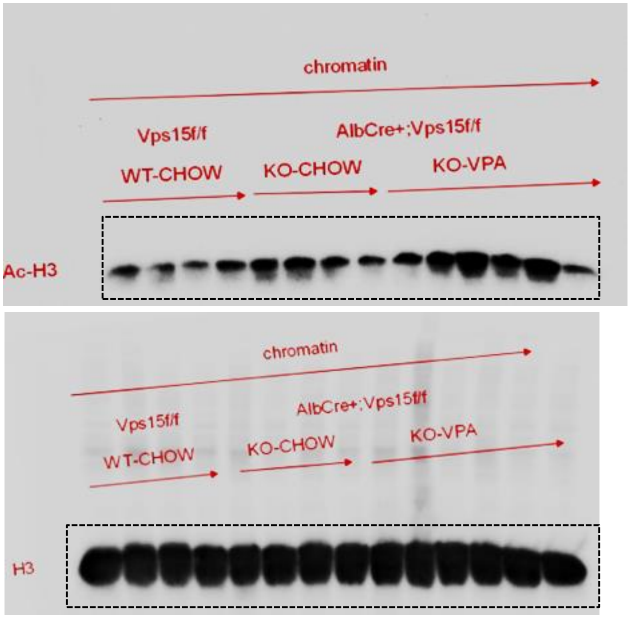

5d

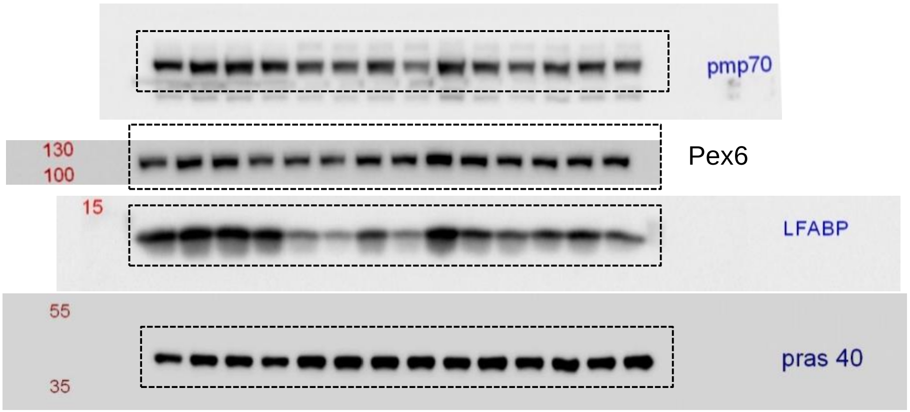

Uncropped blots Figure 6b

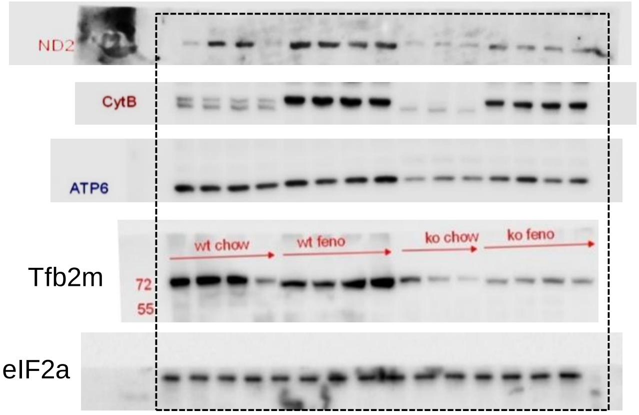

Uncropped blots Figure 7f

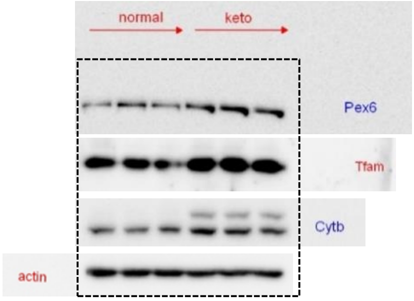

Supplement: Supplementary file 1 — Supplementary Information [file 41467_2019_9598_MOESM1_ESM.pdf]
