# Supplementary material for: The class 3 PI3K coordinates autophagy and mitochondrial lipid catabolism by controlling nuclear receptor PPARα
Source: Nat Commun. 2019 Apr 5;10:1566. doi: 10.1038/s41467-019-09598-9 (PMC6451001; doi:10.1038/s41467-019-09598-9)
Supplement: Supplementary file 3 — Description of Additional Supplementary Files [file 41467_2019_9598_MOESM3_ESM.pdf]

Description of Additional Supplementary Files

File Name: Supplementary Data 1
Description: List of significantly modified genes in livers upon acute deletion of Vps15. The table shows average expression of significantly modified genes (fold change >1.5, P<0.05) in livers of mice transduced with adenoviral vectors expressing Cre recombinase protein to deplete Vps15 or GFP as a control. “Average CRE” and “Average GFP” columns contain the values representing the average gene expression in Cre or GPF group, respectively.
Ratio_Cre vs GFP
represents fold change values over GFP-condition. The statistics is presented as follows: SC - Student t-statistics, BH - Benjamini-Hochberg corrected p-values.

File Name: Supplementary Data 2
Description: Supplementary Data 2A. DAVID functional annotation clustering of genes significantly downregulated in livers upon acute depletion of Vps15 (in relation to Fig.1a). Supplementary Data 2B. DAVID functional annotation clustering of genes significantly upregulated in livers upon acute depletion of Vps15 (in relation to Supplementary Fig.1a). The tables contain the results of DAVID functional annotation clustering and accompanying statistics analyses for the significantly modified genes (fold change >1.5, P<0.05) in livers of mice transduced with adenoviral vectors expressing Cre recombinase protein to deplete Vps15 or GFP as a control. Category - the original database/resource where the term orients; Term represents enriched term associated with gene list; Count - the number of genes involved in the term; % - the percentage of involved genes (involved/total); P-value - a modified Fisher exact p-value, EASE score; List Total - number of genes in gene list; Genes - gene names; Pop Hits - number of genes in the total group of genes assayed that belong to the specific gene category; Pop Total – number of genes in the total group of genes assayed that belong to any Gene Category within the gene categorizing system; Fold Enrichment - measure of magnitude of enrichment; Bonferroni - Bonferroni multiple testing corrected p-value; Benjamini - Benjamini multiple testing corrected pvalue; FDR - false discovery rate.

File Name: Supplementary Data 3
Description: Areas under the peaks for identified metabolites (used for metabolite set enrichment analysis with Metaboanalyst presented on Fig.2a and heatmap on Fig. 2b). Liver tissue of six week old Vps15f/f and AlbCre+;Vps15f/f mice was collected for analyses after six hour fasting. Each column corresponds to an individual Vps15f/f and AlbCre+ ;Vps15f/f animal and contains the values of the area under the peak of indicated metabolite.

File Name: Supplementary Data 4
Description: List of genes expression of which is significantly modified both in livers Vps15 and in PPARa mutants (presented as a heatmap on Fig. 2d). Each column corresponds to an individual animal and contains the values of gene expression in livers of mice. For analyses, the data for all genes, expression of which was significantly modified, was extracted from microarray generated in this study (E-MTAB-7685), PPARa whole body knockout (GSE35015) and PPARa liver-specific knockout (GSE73298). The function of each gene corresponding to Gene Ontology annotation is listed in "GO term" column.

File Name: Supplementary Data 5
Description: Expression level of the genes related to mitochondrial biogenesis and metabolic function in livers of mice that carry a hepatocyte specific deletion of PPARa (presented as a heatmap on Fig. 7a) The table contains values for gene expression levels extracted from microarray experiment performed with liver samples of PPARαf/f and AlbCre+;PPARαf/f mice treated with fenofibrate (GSE73298) or fasted for 24 hours (GSE73299). The genes were selected according to their annotated function for the following categories: PPARa target genes, mitochondrial dynamics genes, mitochondria transcription genes, mitochondria-encoded respiratory chain genes, nuclear coded respiratory chain, TCA and FAO genes. Each column corresponds to individual animal of indicated treatment group.

File Name: Supplementary Data 6
Description: Uncropped blots for Figures 1-7. The uncropped immunoblot panels presented in Figures 1-7 of the manuscript.
