# Supplementary material for: The class 3 PI3K coordinates autophagy and mitochondrial lipid catabolism by controlling nuclear receptor PPARα
Source: Nat Commun. 2019 Apr 5;10:1566. doi: 10.1038/s41467-019-09598-9 (PMC6451001; doi:10.1038/s41467-019-09598-9)
Supplement: Supplementary file 9 — Reporting Summary [file 41467_2019_9598_MOESM9_ESM.pdf]

nature research

Corresponding author(s): Dr. Ganna Panasyuk

Last updated by author(s): 14/03/2019

Reporting Summary

Nature Research wishes to improve the reproducibility of the work that we publish. This form provides structure for consistency and transparency in reporting. For further information on Nature Research policies, see Authors & Referees and the Editorial Policy Checklist.

Statistics

For all statistical analyses, confirm that the following items are present in the figure legend, table legend, main text, or Methods section.

n/a	Confirmed	
☐	☒	The exact sample size (n) for each experimental group/condition, given as a discrete number and unit of measurement
☐	☒	A statement on whether measurements were taken from distinct samples or whether the same sample was measured repeatedly
☐	☒	The statistical test(s) used AND whether they are one- or two-sided<br>*Only common tests should be described solely by name; describe more complex techniques in the Methods section.*
☐	☒	A description of all covariates tested
☒	☐	A description of any assumptions or corrections, such as tests of normality and adjustment for multiple comparisons
☐	☒	A full description of the statistical parameters including central tendency (e.g. means) or other basic estimates (e.g. regression coefficient) AND variation (e.g. standard deviation) or associated estimates of uncertainty (e.g. confidence intervals)
☐	☒	For null hypothesis testing, the test statistic (e.g. F, t, r) with confidence intervals, effect sizes, degrees of freedom and P value noted<br>*Give P values as exact values whenever suitable.*
☒	☐	For Bayesian analysis, information on the choice of priors and Markov chain Monte Carlo settings
☒	☐	For hierarchical and complex designs, identification of the appropriate level for tests and full reporting of outcomes
☒	☐	Estimates of effect sizes (e.g. Cohen's d, Pearson's r), indicating how they were calculated

Our web collection on statistics for biologists contains articles on many of the points above.

Software and code

Policy information about availability of computer code

Data collection	NA
Data analysis	R (http://www.r-project.org/foundation/), Treeview software (http://taxonomy.zoology.gla.ac.uk/rod/treeview.html), MetaboAnalyst 4.0 (https://www.metaboanalyst.ca/).

For manuscripts utilizing custom algorithms or software that are central to the research but not yet described in published literature, software must be made available to editors/reviewers. We strongly encourage code deposition in a community repository (e.g. GitHub). See the Nature Research guidelines for submitting code & software for further information.

Data

Policy information about availability of data

All manuscripts must include a data availability statement. This statement should provide the following information, where applicable:

- Accession codes, unique identifiers, or web links for publicly available datasets
- A list of figures that have associated raw data
- A description of any restrictions on data availability

Previously published microarray data were reanalysed from the GEO data sets (GSE35015, GSE73298, GSE73299).
All data are available from the corresponding author upon reasonable request. The information and requests for resources and materials should be directed and will be fulfilled by Dr. Ganna Panasyuk (ganna.panasyuk@inserm.fr). The source data underlying all figures in the main text of the manuscript are provided as a Source Data file. The source data for microarray analyses in Vps15 depleted liver are deposited in ArrayExpress database E-MTAB-7685.

Field-specific reporting

Please select the one below that is the best fit for your research. If you are not sure, read the appropriate sections before making your selection.

☒ Life sciences ☐ Behavioural & social sciences ☐ Ecological, evolutionary & environmental sciences

For a reference copy of the document with all sections, see nature.com/documents/nr-reporting-summary-flat.pdf

Life sciences study design

All studies must disclose on these points even when the disclosure is negative.

Sample size	No statistical method was used to predetermine sample size. Experiments were independently repeated at least twice as indicated, and mean and the standard error from the mean were calculated. In studies with animals, the animal numbers were chosen to reflect the expected magnitude of response taking into account the variability observed in previous experiments.
Data exclusions	No data were excluded from the analyses.
Replication	Experiments were carried out in biological and/or technical replicates as indicated in the results part (text and figure legends). The reproducibility of the experimental findings were verified by performing additional independent experiments (at least two) or by having several technical replicates (as described in the figure legends). All attempts at replication were confirmed to be successful.
Randomization	Randomization was not relevant in this study as there was no comparison of cohorts.
Blinding	Investigators were blinded to group allocation for histology analyses, immunofluorescent microscopy, electron microscopy quantification and PLA assay quantification. All other experiments were performed in non-blinded manner.

Reporting for specific materials, systems and methods

We require information from authors about some types of materials, experimental systems and methods used in many studies. Here, indicate whether each material, system or method listed is relevant to your study. If you are not sure if a list item applies to your research, read the appropriate section before selecting a response.

Materials & experimental systems

n/a	Involved in the study	
☐	☒	Antibodies
☐	☒	Eukaryotic cell lines
☒	☐	Palaeontology
☐	☒	Animals and other organisms
☒	☐	Human research participants
☒	☐	Clinical data

Methods

n/a	Involved in the study	
☒	☐	ChIP-seq
☒	☐	Flow cytometry
☒	☐	MRI-based neuroimaging

Antibodies

Antibodies used	Vps15 (1:1000, Abnova, H00030849-M03; 1:1000, Genetex, GTX108953), p62 (1:1000, Abnova, H00008878-MO1), β-actin (1:5000, Sigma, A5316), Tubulin (1:1000, Sigma, T9026), HA (1:1000, 1:200 for IF, Sigma, H9658), Pmp70 (1:500, Sigma, SAB4200181), β-catenin (1:500, BD Biosciences, 610153), Cytochrome C (BD Biosciences, 556432), Pras40 (1:1000, Cell Signaling, 2691), Ulk1(1:1000, Cell signaling, 8054S), Lamin A/C (1:1000, Cell Signaling, 2032), NCoR1 (1:1000, Cell Signaling, 5948), Hdac3 (1:1000, Cell Signaling, 85057), H3K27Ac (1:1000, Cell Signaling, 8173), H3 (1:1000, Cell Signaling, 4499), Ulk1(1:1000, Cell signaling, 8054S), Ubiquitin (1:1000, Cell Signaling, 3936), pS616 Drp1 (1:1000, Cell Signaling, 3455), Drp1(1:1000, Cell Signaling, 8570), LC3xp (1:500, Cell Signaling, 3868), LC3 (1:1000, NanoTools, 0231-100/LC3-3-5-5F10), eIF2α (1:1000, Santa Cruz, sc-11386), PPARα (1:500, Santa Cruz, sc-398394, sc-9000), Tfb2m (1:500, Santa Cruz, sc-517095), GAPDH (1:1000, Santa Cruz, SC-25778), Nrf2 (1:1000, Santa Cruz, sc-13032), Parkin (1:1000, Santa Cruz, sc32282), GST (1:1000, Santa Cruz, sc-459), Pex6 (1:500, Santa Cruz, sc-271813), Fabp1 (1:500, Santa Cruz, sc-50380), NCoR1 (1:500, sc-515934, Santa Cruz), Cre (1:1000, GTX127270, GeneTex), Tom40 (1:1000, ProteinTech, 18409-1-AP), ND2 (1:500, Proteintech, 19704-1-AP), Cytb (1:500, Proteintech, 55090-1-AP), ATP6 (1:500, Proteintech, 55313-1-AP), PGC1α (1:1000, Proteintech, 66369-1-Ig), Hdac1 (1:1000, Thermo, PA1-860), Lamp1 (1:1000, Abcam, ab24170), Lamp2 (1:1000, Abcam, ab13524), GFP (1:1000, Clontech, 8362-1), Huwe1 (1:1000, Cell Signaling, 5695).
Validation	Validation statements for all the antibodies used in the study are available at the websites of the respective commercial providers.

Eukaryotic cell lines

Policy information about cell lines

Cell line source(s)	HEK293T cells, acquired from ATCC.
Authentication	HEK293T cell line was obtained from original source and was not further authenticated.
Mycoplasma contamination	HEK293T cells were routinely bi-weekly tested for potential contaminations with mycoplasma using commercial PCR Mycoplasma Detection Kit (ABM, #G-238). All tests were negative.
Commonly misidentified lines (See ICLAC register)	Does not apply.

Animals and other organisms

Policy information about studies involving animals; ARRIVE guidelines recommended for reporting animal research

Laboratory animals	AlbCre+;Vps15f/f (Nemazanyy et al., Nature Comm, 2015, derived from the strain #022624, available from the Jackson Laboratory, USA). Male mice (6-8 week old) were used for the experimentation. All animals used in the study were fed ad libitum standard chow diet (Teklad global protein diet; 20% protein, 75% carbohydrate, 5% fat) and kept under 12h/12h (8am/8pm) light on/off cycle. Animals were sacrificed between 2-4pm (ZT6-ZT8) unless indicated. All animal studies were performed by authorized users in compliance with ethical regulations for animal testing and research.
Wild animals	The study did not involve wild animals.
Field-collected samples	The study did not involve samples collected in the field.
Ethics oversight	The study was approved by the Direction Départementale des Services Vétérinaires, Préfecture de Police, Paris, France (authorization number 75-1313) and the ethical committee of Paris Descartes University (authorization number 17-052).

Note that full information on the approval of the study protocol must also be provided in the manuscript.
